# High frequency of *CRB1* mutations as cause of Early-Onset Retinal Dystrophies in the Spanish population

**DOI:** 10.1186/1750-1172-8-20

**Published:** 2013-02-05

**Authors:** Marta Corton, Sorina D Tatu, Almudena Avila-Fernandez, Elena Vallespín, Ignacio Tapias, Diego Cantalapiedra, Fiona Blanco-Kelly, Rosa Riveiro-Alvarez, Sara Bernal, Blanca García-Sandoval, Montserrat Baiget, Carmen Ayuso

**Affiliations:** 1 Department of Genetics, IIS - Fundación Jiménez Díaz, Madrid, Spain; 2 Department of Ophthalmology, Fundación Jiménez Díaz, Madrid, Spain; 3 Department of Genetics, Instituto de Investigación Hospital de la Santa Creu i Sant Pau, Barcelona, Spain; 4 Centre for Biomedical Network Research on Rare Diseases (CIBERER), ISCIII, Valencia, Spain

**Keywords:** Leber congenital amaurosis, Early-onset retinitis pigmentosa, *CRB1*, Homozygosity mapping, High resolution melting

## Abstract

**Background:**

*CRB1* mutations are reported as cause of severe congenital and early-onset retinal dystrophies (EORD) with different phenotypic manifestations, including Leber congenital amaurosis (LCA), retinitis pigmentosa (RP) and cone-rod dystrophies. Comprehensive mutational scanning of the whole gene has been only performed in few cohorts, mainly in LCA patients. Here, we aimed investigating the real prevalence of *CRB1* mutations in the Spanish population by extensive screening of *CRB1* mutations in a large cohort of LCA and EORP cases.

**Methods:**

This report integrates data from previous studies on *CRB1* defects in our Spanish cohort of LCA and early-onset RP (EORP) with new findings from a comprehensive mutational screening of the whole gene. The molecular tools used include mutation genotyping arrays, whole-genome homozygosity mapping, an optimized high-resolution melting (HRM) analysis and Sanger sequencing.

**Results:**

A large clinically well-characterized cohort of 404 Spanish cases was studied, 114 of which suffered from LCA and 290 from EORP. This study reveals that 11% of Spanish patients carried mutations in *CRB1*, ranging from 9% of EORP to 14% of LCA cases. More than three quarters of the mutations identified herein have been first described in this Spanish cohort, 13 of them are unreported new variants and 13 had been previously reported in our previous studies.

**Conclusions:**

This work provides a wide spectrum of *CRB1* mutations in the Spanish EORD patients and evidences the major role of *CRB1* as causal gene in the Spanish EORP patients. It is noteworthy that a high rate of private mutations only described in our cohort has been found so far. To our knowledge, this study represents the most complete mutational screening of *CRB1* in a Spanish LCA and EORP cohort, allowing us to establish gene-specific frequencies and to provide a wide spectrum of *CRB1* mutations in the Spanish population.

## Background

Mutations in the human *Crumbs homolog 1* (*CRB1,* MIN #604210) gene lead to severe congenital and early-onset retinal dystrophies (EORD) with a wide range of clinical manifestations including Leber congenital amaurosis (LCA, MIN #613835), early-onset Retinitis Pigmentosa (EORP) with or without preserved para-arteriolar retinal pigment epithelium (PPRPE; MIN #600105), Coats-like exudative vasculopathy, and juvenile cone-rod dystrophies [1-5]. The prevalence of *CRB1* mutations varies widely depending on the clinical phenotype and the cohort studied, ranging between 7% in EORD patients [6], 10% in LCA patients [7], 31% in RP patients with Coats-like exudative vasculopathy and 66% in RP patients with PPRPE [8]. *CRB1* mutations are rare (2%) in RP without signs of PPRPE or Coats-like exudative vasculopathy [8].

The *CRB1* gene, located in 1q31, contains 12 exons and an alternative splicing at the 3’ end, encoding two different isoforms of 1,376 and 1,406 amino acids [9]. Both proteins have a signal peptide sequence, 19 epidermal growth factor (EGF)-like domains, 3 laminin A globular (AG)-like domains, and the longer isoform also contains additional transmembrane and cytoplasmatic domains [9]. In total, over 150 different mutations have been identified, mainly located in the extracellular domain [10], and so, suggesting to play an important role probably by interacting with unknown proteins [11]. *CRB1* is expressed in retina and brain and is highly homologous to the *Drosophila melanogaster* Crumbs (crb) protein [1,12]. In the mouse retina, *CRB1* is located in the apical region of retinal pigment epithelial (RPE) cells, rod and cone photoreceptor cells and Muller glial cells [13]. In this way, the *Drosophila* Crumbs loss-of-function (LOF) mutant leads to similar photoreceptor defects to those observed in patients carrying *CRB1* mutations [11]. Both human and fly proteins seem to play an essential role in photoreceptor morphogenesis, including maintenance of the polarity of RPE cells [13,14].

Preliminary studies in the Spanish population have shown *CRB1* as the main mutated gene in LCA patients (17%) [15] but it seems to explain only about 2% of EORP [16]. In both studies, a commercial APEX (Arrayed Primer EXtension)-based microarray for LCA or arRP was used to genotype previously known *CRB1* mutations. Therefore it is likely that the real impact of *CRB1* mutations is underestimated. Some Spanish cases had previously studied for *CRB1* mutations using several mutational scanning methods such as single-strand conformational polymorphism analysis (SSCP) or denaturing high-performance liquid chromatography (dHPLC) [5,15], which are expensive and technically time-consuming. By contrast, the recently developed high resolution melting (HRM) analysis allows a simple, semi-automated, and cost-effective detection of single-base substitutions and small insertions/deletions [17].

Here, with the aim of investigating the prevalence of *CRB1* mutations in the Spanish population, we performed a comprehensive screening of *CRB1* mutations in a large cohort of Spanish LCA/EORP patients using different indirect and direct molecular approaches in a sequential way. HRM scanning and Sanger sequencing were mainly used to find novel *CRB1* disease alleles in patients in whom no mutation was identified by a preliminary microarray genotyping.

## Methods

### Participants and clinical evaluation

Patients diagnosed with LCA or EORP were recruited from Fundación Jiménez Díaz Hospital (FJD, Madrid, Spain) from 1990 to 2011. Informed consent was obtained from all subjects prior to their participation in this study. All procedures were reviewed and approved by the Ethics Committee of the hospital and adhered to the tenets of the Declaration of Helsinki.

Clinical diagnosis of RD was based on measurements of visual acuity and visual field tests, fundus examination and electroretinogram (ERG) responses. Diagnostic criteria of LCA included: 1) severe visual loss from birth or before 1 year of age; 2) congenital nystagmus; 3) sluggish or absent pupillary responses and 4) non-recordable or significantly reduced ERG. Diagnostic criteria of EORP included poor night vision and/or peripheral visual loss in childhood, with poor visual acuity and visual field loss in advanced stages of the pathology. Patients with family pedigree compatible with autosomal dominant or X-linked inheritance or with any systemic sign underlying syndromic forms of retinal dystrophy were excluded.

A total of 404 unrelated Spanish families with autosomal recessive or isolated retinal dystrophy were selected: 114 families with LCA and 290 families with EORP. Genomic DNA was obtained from peripheral blood samples from FJD Biobank using an automated DNA extractor (BioRobot EZ1 Qiagen, Hilden, Germany) following the manufacturer instructions.

Previously, 23 of these 404 patients were studied for *CRB1* mutations by SSCP as described by Bernal et al. [5]. Two of them were found to carry pathogenic variants in *CRB1* and were included in the summaries presented herein.

One hundred sixty-five healthy unrelated Spanish individuals without personal or familial history of retinal dystrophy were screened as controls to evaluate the frequency of the novel variants.

### Mutation detection by APEX microarray

Index cases of the 404 families were screened for known mutations to cause LCA or autosomal recessive RP (arRP) using a commercial genotyping chip based on APEX technology (LCA or ARRP chip, Asper Ophthalmics, Tartu, Estonia). Both chips include 114 known mutations and rare variants in the *CRB1* gene. All variants identified by the previous methodology were further validated by Sanger sequencing and, whenever DNA was available, familial segregation was verified. In cases when only one allele was identified in the *CRB1* gene, microsatellite analysis and/or direct mutational screening by dHPLC, HRM or Sanger sequencing was used in order to detect the presence of a second pathogenic variant.

### Mutation scanning by dHPLC and HRM analysis

Exons and exon-intron boundaries of *CRB1* (RefSeq NM_201253) were analysed using 25 oligonucleotide primer pairs designed by Hanein et al., 2004 or specifically designed using Primer3 software http://primer3.wi.mit.edu/, as detailed in Additional file 1: Table S1.

After standard PCR amplification, dHPLC analysis was performed with the WAVE DNA fragment analysis system (Transgenomic) as previously reported [5,15,18].

*A* HRM approach was specifically developed and optimised for mutational scanning of the *CRB1* gene. Real-time PCR and HRM were consecutively done on a LightCycler 480 Real-Time PCR System (Roche) in one single run, and all reactions were performed in duplicate. PCR amplifications were done in the presence of 2.5 mmol/L MgCl_2_ according to the protocols provided by Roche. PCR conditions were: 95°C for 10 minutes, followed by 45 cycles of pre-incubation at 95°C for 20 seconds, annealing for 20 seconds at the indicated temperature in Additional file 1: Table S1 and extension at 72°C for 20 seconds. After amplification, PCR products were denatured at 95°C for 1 minute and cooled down to 40°C to allow hetero-duplex formation. The final HRM step was performed from 40°C to 95°C with an increase of 1°C/s with 25 acquisitions/°C. The HRM curve analysis was performed using the LightCycler 480 Gene Scanning Software (Roche). Melting curves were normalized, temperature-adjusted and finally, a difference plot was generated.

For methodological optimization, HRM analysis was further applied on both control samples and mutated patients carrying previously identified *CRB1* genetic variants by Sanger sequencing. As the majority of known alleles in *CRB1* are located in exons 2, 7 and 9, we first analysed 5 different variants in these exons by HRM: c.498_506del on amplicon 2b, c.2244-47delATC on amplicon 7a, c.2290C>T and c.2307C>T on amplicon 7b; c.2843 G>A on amplicon 9a. Melting curves generated from these amplicons allowed an easy discrimination of all these variants, except the change in exon 9a coding for the most prevalent mutation, p.Cys948Tyr that could not be detected (Additional file 2: Figure S1).

Two hundred twenty-five index cases were screened by HRM analysis. All amplicons were further sequenced to discriminate not only disease-causing mutations and benign polymorphisms but also false positive and false negative HRM profiles. Sensitivity and specificity of HRM scanning to identify *CRB1* mutations were also calculated using Sanger sequencing as gold standard.

### Sanger sequencing

PCR products were enzymatically purified using ExoSAP-it (USB, Affymetrix) and sequenced on both strands using the Big Dye Terminator Cycle Sequencing Kit v3.1 Kit (Applied Biosystems). The sequence products were purified on a 96-well multiscreen filter plate (Montage SEQ96 Sequencing Reaction Cleanup Kit, Millipore, Bedford, MA) and resolved on an automated sequencer (ABI 3130xl Genetic Analyzer, Applied Biosystems).

### Indirect analysis

Microsatellite analysis was performed in 80 families using flanking polymorphic markers (TEL-D1S408, D1S2757, D1S2816, D1S1660*-*CEN), as previously described [15]. After carrying out the PCR amplification, fluorescent-labelled products were analysed in an automatic sequencer (3130xl ABI Prism, Applied Biosystems, CA, USA). Whole genome homozygosity mapping was performed in 66 consanguineous and non-consanguineous families using high-resolution commercial SNP arrays from Affymetrix (Genome Wide Human SNP array 6.0 and GeneChip Human Mapping 500 K Array Set) or Illumina (HumanLinkage V Panel Set or Omni Whole Genome arrays HumanCytoSNP-12). Arrays were processed according to the manufacturer protocols. Affymetrix genotyping services were provided by the Spanish National Genotyping Center (CEGEN-ISCIII)”. Homozygosity regions were calculated using using the Linkage Disequilibrium - Hidden Markov Model algorithm (LD-HMM) [19] through the dCHIP software [20].

### MLPA Analysis

Multiplex Ligation-dependent Probe Amplification (MLPA) was performed to discard large deletions or duplications in patients carrying only one *CRB1* allele after the above molecular screening. The commercial P221 LCA mix-1 SALSA MLPA kit (MRC Holland, Amsterdam, Netherlands) that contains specific probes for all exons of the *CRB1* gene, except for exon 11, was used according to the manufacturer recommendations. The amplified fragments were separated by capillary electrophoresis using an ABI 3130xl automatic analyser (Applied Biosystems).

### Assessment of the pathogenicity of new and unclassified variants

Pathogenicity of unreported variants was established by the following criteria: 1) co-segregation in the family, 2) absence in 165 Spanish healthy control individuals, 3) amino acid conservation for missense mutations, 4) pathogenicity prediction with *in silico* tools, and 5) presence of a second pathogenic allele. These criteria were applied to the new variants found in this study as well as for previously unclassified missense variants.

Amino acid conservation was determined using 17 orthologs of the *CRB1* protein belonging to different evolutionary branches (Chimpanzee, Marmoset, Rhesus Macaque, Orangutan, Gibbon, Cow, Rat, Mouse, Rabbit, Dog, Horse, Wild boar, Opossum, Turkey, Lizard, Frog and Zebrafish). BLINK tool (http://www.ncbi.nlm.nih.gov/sutils/blink.cgi?pid=6014482) and Jalview Alignment Editor program (http://www.jalview.org/) were used to analyse the multiple sequence alignments. If a residue did not change throughout the species it was considered “highly conserved”, if a residue was present in less than 3 species it was considered “moderately conserved”, if it was present in 3–5 ortholog proteins, it was considered “weakly conserved”, otherwise it was classified as “non conserved”. Deleterious effects of amino acid substitutions on protein function were assessed using different bioinformatic programs, including Align-GVGD (http://agvgd.iarc.fr/agvgd_input.php), PolyPhen-2 (Polymorphism Phenotyping v2) (http://genetics.bwh.harvard.edu/pph2/), SIFT (http://sift.jcvi.org/) and Protein Variation Effect Analyzer (PROVEAN) (http://provean.jcvi.org/index.php). Splice-site mutations were analysed with the Human Splicing Finder (HSF) tool (http://www.umd.be/HSF/).

## Results

DNA samples from 404 Spanish index cases with LCA or EORP were first analysed using the LCA/ARRP chips in order to identify known mutations, followed by further screening of *CRB1* mutations using several indirect and direct strategies, such as homozygosity mapping, HRM analysis and Sanger sequencing, as summarized in Table 1.

**Table 1:** General description of patients and molecular tools included in this study

**Families in study**	**APEX microarray**			**Indirect studies**		**SSCP **^**&**^	**HRM**			**Sanger sequencing**	**Total families characterized**	
	**CRB1 2 alleles**	Second allele by whole CRB1 analysis *	**Total characterized**	**Haplotypes**	**IBD mapping**	**CRB1 2 alleles**	**CRB1 2 alleles**	**CRB1 1 allele**	Second allele by Sanger sequencing *	***2 alleles***	***CRB1 mutations***	***Mutations in another gene ***^***#***^
**114 LCA**	8 /114	4 / 8 ^**a**^	**12** (10%)	0 / 20	**1 **^**b**^/43	**1 / 3**	**1 / 32**	1 / 32	0 / 1	**1/51**	**16** (14%)	32 (28%)
**290 EORP**	6 /290	15 /25 ^**a**^	**21** (7%)	0 / 60	0 / 23	**1 / 20**	0 / 193	1 / 193	**1** / 1 ^**c**^	**4 **^**d**^/ 209	**27** (9%)	59 (20%)
**404 Total**	14 / 404	19 /33	**33** (8%)	0 / 80	**1** / 66	**2** / 23	**1** / 225	2 / 225	**1** / 2	**5**/ 260	**43** (11%)	91 (22%)

Overview of the sequential steps performed during the mutational *CRB1* analysis in a cohort of LCA and EORP Spanish patients was reflected. The number of cases characterized and studied is outlined for each molecular tool. APEX: Arrayed primer extension; IBD: Inherited-by-descent; SSCP: single-strand conformation polymorphism analysis; HRM: High resolution melting analysis. * A direct mutational scanning of *CRB1* was performed using dHPLC, HRM or Sanger sequencing in patients with a first allele identified by APEX microarray. ^&^ SSCP findings were reported by Bernal S. et als, 2003. ^#^ Mutations in another gene were found by APEX microarray, whole-genome homozygosity maping, whole exome sequencing or targeted NGS (data not shown).

^a^ A second CRB1 allele was not found in 14 patients: 2 carried a known frameshift mutation and 12 carried a uncertain or very unlikely missense variant.

^b^ A 5 Mb-homozygous region involving *CRB1* was identified in an endogamic family and a homozygous known mutation was further found by Sanger sequencing. This mutation represents a false negative of the previously LCA chip analysis.

^c^ A second allele (c.1702C>T) was further found by Sanger sequencing, representing a false negative of the HRM analysis.

^d^ Two heterozygous transitions (c.2291 G>A and c.4168C>T) were found in one patient that previously showed normal melting curves in the HRM analysis thus, representing false negatives.

Two causative *variants* were found in the 11% (43 / 404) of our Spanish patients (Table 2). Considering the two different phenotypes studied, 14% of LCA and 9% of early-onset RP patients carried *CRB1* mutations (Additional file 2: Figure S2, S3 and S4). A pathogenic *CRB1* allele was also identified in 2 additional patients but a second variant could not be identified by whole screening of this gene by Sanger sequencing and MLPA (Table 2). Heterozygous missense variants with very unlikely pathogenic implication (Additional file 1: Table S2 and Additional file 2: Figure S5) were found in other 13 patients.

**Table 2:** **Patients carrying *CRB1 *mutations identified in this study**

**Family**			**Allele 1**			**Allele 2**	**References**	**Methods**	**Phenotype**
	**Exon**	**Nucleotide Change**	**AA Change**	**Exon**	**Nucleotide Change**	**AA Change**			
LCA-0010	2	**c.481dupG**	p.Ala161Glyfs*8	2	**c.481dupG**	p.Ala161Glyfs*8	[5]	chip	LCA
RP-0091	2	**c.481dupG**	p.Ala161Glyfs*8		?	?	[5]	chip + seq	EORP
RP-1426	2	**c.498_506del**	**p.Ile167_Gly169del**	2	**c.498_506del**	**p.Ile167_Gly169del**	[21]	seq	EORP
RP-1611	2	**c.498_506del**	**p.Ile167_Gly169del**	5	**c.1147_1156del**	p.Cys383Serfs*66	[21], Novel	chip + seq	EORP
RP-2004	2	**c.498_506del**	**p.Ile167_Gly169del**	7	c.2234C > T	p.Thr745Met	[21][1]	chip + seq	EORP
RP-0745	2	**c.498_506del**	**p.Ile167_Gly169del**	7	c.2290C > T	p.Arg764Cys	[21][1]	chip + HRM	EORP
RP-0243	2	**c.498_506del**	**p.Ile167_Gly169del**	8	c.2688 T > A	p.Cys896*	[21][22]	chip + dHPLC	EORP
MD-0092b	2	**c.498_506del**	**p.Ile167_Gly169del**	9	c.2843G > A	p.Cys948Tyr	[21][1]	chip + HRM	EORP
LCA-0019	2	c.613_619del	p.Ile205Aspfs*13	7	**c.2227delG**	p.Val743Serfs*11	[2][23]	chip + dHPLC	EORP
LCA-0051	2	c.613_619del	p.Ile205Aspfs*13	9	c.2843G > A	p.Cys948Tyr	[2][1]	chip	EORP
LCA-0063	2	c.613_619del	p.Ile205Aspfs*13	11	c.4005 + 1G > A	Splicing	[2][22]	chip	EORP
RP-1311	2	c.613_619del	p.Ile205Aspfs*13		?	?	[2]	chip + seq	EORP
LCA-0032	6	**c.1604 T > C**	**p.Leu535Pro**	9	c.2843G > A	p.Cys948Tyr	[15][1]	chip + seq	LCA
LCA-0099	6	**c.1690G > T**	**p.Asp564Tyr**	8	c.2688 T > A	p.Cys896*	[15][22]	chip	LCA
LCA-0038b	6	**c.1690G > T**	**p.Asp564Tyr**	9	**c.3002 T > A**	**p.Ile1001Asn**	[15]	seq	EORP
RP-1535	6	**c.1690G > T**	**p.Asp564Tyr**	9	**c.3014A > T**	**p.Asp1005Val**	[15], Novel	chip + seq	EORP
RP-1504	6	**c.1702C > T**	**p.His568Tyr**	12	c.4142C > T	p.Pro1381Leu	Novel [6]	HRM + seq	EORP
RP-1586	7	c.2234C > T	p.Thr745Met	7	c.2234C > T	p.Thr745Met	[1]	chip	EORP
RP-1017	7	c.2234C > T	p.Thr745Met	7	**c.2416G > T**	p.Glu806*	[1] Novel	chip + seq	EORP
MD-0643	7	c.2234C > T	p.Thr745Met	9	c.2843G > A	p.Cys948Tyr	[1]	chip	EORP
RP-0561	7	c.2234C > T	p.Thr745Met	11	**c.3988G > T**	p.Glu1330*	[1][24]	chip + dHPLC	EORP
LCA-0011	7	**c.2244_47delATC**	**p.Ser749del**	9	c.2843G > A	p.Cys948Tyr	[5][1]	chip	LCA
RP-1779	7	c.2290C > T	p.Arg764Cys	9	**c.3299 T > C**	**p.Ile1100Thr**	[1][5]	chip	EORP
RP-0487	7	c.2290C > T	p.Arg764Cys	11	**c.3988G > T**	p.Glu1330*	[1][24]	chip + dHPLC	EORP
RP-1689	7	**c.2291G > A**	**p.Arg764His**	12	**c.4168C > T**	p.Arg1390*	Novel	seq	EORP
LCA-0060	7	**c.2309G > T**	**p.Gly770Val**	8	**c.2805dupA**	p.His935Glnfs*13	Novel	HRM	LCA
LCA-0017	7	c.2401A > T	p.Lys801*	7	c.2401A > T	p.Lys801*	[4]	IBD mapping	LCA
RP-0280	7	**c.2465G > A**	p.Trp822*	9	c.2843G > A	p.Cys948Tyr	[15][1]	chip + dHPLC	EORP
LCA-0004	8	c.2688 T > A	p.Cys896*	9	c.2843G > A	p.Cys948Tyr	[22][1]	chip	LCA
LCA-0038a	8	c.2688 T > A	p.Cys896*	9	**c.3002 T > A**	**p.Ile1001Asn**	[22][15]	chip + dHPLC	LCA
LCA-0050	9	c.2843G > A	p.Cys948Tyr	9	c.2843G > A	p.Cys948Tyr	[1]	chip	LCA
RP-0069a	9	c.2843G > A	p.Cys948Tyr	9	c.2843G > A	p.Cys948Tyr	[1]	SSCP	LCA
MD-0092a	9	c.2843G > A	p.Cys948Tyr	9	c.2843G > A	p.Cys948Tyr	[1]	chip	EORP
MD-0351	9	c.2843G > A	p.Cys948Tyr	9	**c.3157 A > G**	**p.Met1053Val**	[1] Novel	chip + HRM	EORP
RP-1625	9	c.2843G > A	p.Cys948Tyr	9	**c.3157A > G**	**p.Met1053Val**	[1] Novel	chip + HRM	EORP
LCA-0028	9	c.2843G > A	p.Cys948Tyr	9	**c.3299 T > C**	**p.Ile1100Thr**	[1][5]	chip	LCA
RP-1558	9	c.2843G > A	p.Cys948Tyr	9	**c.3607G > T**	p.Glu1203*	[1] Novel	chip + seq	EORP
LCA-0027	9	c.2843G > A	p.Cys948Tyr	11	**c.3988G > T**	p.Glu1330*	[1][24]	chip + seq	LCA
LCA-0038c	9	**c.3002 T > A**	**p.Ile1001Asn**	9	**c.3482A > G**	**p.Tyr1161Cys**	[15] [25]	seq	EORP
LCA-0101	9	**c.3152G > A**	p.Trp1051*	11	**c.4000delG**	p.Val1334Trpfs*7	Novel	seq	LCA
RP-0069b	9	**c.3299 T > C**	**p.Ile1100Thr**	9	c.2843G > A	p.Cys948Tyr	[5][1]	SSCP	EORP
RP-0025	9	**c.3299 T > C**	**p.Ile1100Thr**	9	**c.3299 T > C**	**p.Ile1100Thr**	[5]	chip	EORP
RP-1212	9	**c.3299 T > C**	**p.Ile1100Thr**	9	**c.3299 T > C**	**p.Ile1100Thr**	[5]	chip	EORP
RP-1615	9	**c.3299 T > C**	**p.Ile1100Thr**	9	**c.3749 + 1_3749 + 2delGT**	**Splicing**	[5] Novel	chip + seq	EORP
RP-1440	10	**c.3878 + 2insT **^**a**^	**Splicing**	8	**c.2696G > C**	**p.Gly899Ala**	Novel	chip + HRM	EORP

Mutations in bold correspond to variants first described in our cohort. Nucleotide numbering reflects cDNA in the reference sequence NM_201253.1, according to journal guidelines (http://www.hgvs.org/mutnomen). The initiation codon is codon 1. Different molecular approaches were used to identify both pathogenic alleles. C: APEX microarray, S: Sanger sequencing, D: dHPLC, denaturing high-performance liquid chromatography analysis, H, HRM: high-resolution melting analysis, IBD: Inherited-by-descent mapping, performing using whole-genome SNP arrays; ?: Second allele not found after Sanger sequencing and MLPA analysis;

^a^ Unexpected signal on APEX array was detected at the interrogated nucleotide c.3878 + 2 further confirming as a novel heterozygous insertion by Sanger sequencing.

^&^T allele is paternally inherited and c.2805insA is a *de novo* mutation.

The molecular analysis of *CRB1* revealed 34 different mutations in 45 Spanish LCA/EORP patients, as detailed in Additional file 1: Table S3. Most of the disease alleles were in exons 9 (43%), 7 (19%) and 2 (16%). Therefore, 76% (26 / 34) of the mutations, representing 55% of the total *CRB1* alleles, have been first described in this cohort (Table 3). Compound heterozygous *CRB1* mutations were found in all cases except for 9 families with known inbreeding or endogamic history. The most frequent mutation representing the 22% of *CRB1* alleles was p.Cys948Tyr identified in 16 families. This mutation was in heterozygous state in all cases except for 3 homozygous patients. The variants p.Ile1100Thr and c.498_506del that were first identified in our cohort, are also quite common with a frequency of 9 and 8%, respectively. Similarly, other mutations such as p.Asp564Tyr, p.Ile1001Asn and p.Glu1330* were present in more than one family (Additional file 1: Table S3).

**Table 3:** **List of novel *CRB1 *likeky pathogenic variants identified in the studied cohort**

**Nucleotide change**	**AA change**	**Protein**	**Conservation**	**SIFT score**	**Polyphen score**	**GD-GV score**	**PROVEAN score**	**Pathogenicity**
		**Domain**						
***Missense***								
c.1604 T > C	p.Leu535Pro	Lam AG1	HC	0	1	Class C65	-6.5	Likely
c.1690G > T	p.Asp564Tyr	Lam AG1	HC	0	1	Class C65	-8.2	Likely
c.1702C > T	p.His568Tyr	Lam AG1	HC	0	0.999	Class C65	-5.0	Likely
c.2291G > A	p.Arg764His	Lam AG2	NC	0.06	0.012	Class C0	-2.3	Uncertain ^a^
c.2309 G > T	p.Gly770Val	Lam AG2	HC	0	1	Class C65	-8.2	Likely
c.2696 G > C*	p.Gly899Ala	EGF13	HC	0.1	0.996	Class C0	-4.5	Likely
c.3002A > T	p.Ile1001Asn	Lam AG3	MC	0.0	0.850	Class C45	-5.5	Likely
c.3014 A > T	p.Asp1005Val	Lam AG3	WC	0.02	0.996	Class C15	-5.0	Likely
c.3157 A > G	p.Met1053Val	Lam AG3	HC	0	0.994	Class C15	-2.2	Likely
c.3299 T > C	p.Ile1100Thr	Lam AG3	C	0	0.977	Class C25	-3.6	Likely
c.3482A > G	p.Tyr1161Cys	EGF15	MC	0	0.999	Class C15	-6.1	Likely
***Nonsense***								
c.2416G > T	p.Glu806*	Lam AG2	Protein truncation, NMD					
c.2465G > A	p.Trp822*	Lam AG2	Protein truncation, NMD					
c.3152G > A	p.Trp1051*	Lam AG3	Protein truncation, NMD					
c.3607 G > T	p.Glu1203*	EGF16	Protein truncation, NMD					
c.3988G > T	p.Glu1330*	EGF19	Protein truncation, NMD					
c.4168C > T	p.Arg1390*	C	Protein truncation					
***Frameshift indels***								
c.481dupG	p.Ala161Glyfs*8	EGF4	Protein truncation, NMD					
c.1147_1156del	p.Cys383Serfs*66	EGF9	Protein truncation, NMD					
c.2227delG	p.Val743Serfs*11	Lam AG2	Protein truncation, NMD					
c.2805dupA	p.His935Glnfs*13	EGF14	Protein truncation, NMD					
c.4000delG	p.Val1334Trpfs*7	EGF19	Protein truncation, NMD					
***Splicing***								
c.3749 + 1_3749 + 2del	-	EGF17	Splicing defect, NMD					
c.3878 + 2insT	Splicing	EGF18	Splicing defect, NMD					
**In-frame indel**								
c.2244_47delATC	p.Ser749del	Lam AG2	WC				-11.453	Likely
c.498_506del	p.Ile167_Gly169del	EGF4	Gly167: MC				-14.258	Likely

Nucleotide numbering is based on RefSeq DNA accession number NM_201253.1. Lam AG: Laminin AG-like domain, EGF: EGF-like domain, C: cytoplasmatic domain. Conservation of the amino acid substituted or deleted in 17 species was detailed. HC: Highly conserved, MC: Moderately conserved, WC: Weakly conserved, and NC: non-conserved residue. The amino acid substitution is predicted damaging by if the SIFT score is < = 0.05 and PROVEAN scores is < -2.5. Polyphen predict a non-synonymous variant as benign, possibly damaging, or probably damaging, if score is < 0.2, between 0.2 and 0.85 or > 0.85. GV: Grantham Variation; GD: Grantham distribution. Class C65: most likely pathogenic, Class C0: less likely pathogenic.

^a^ This variant has been considered as likely pathogenic despite of poor conservation and less likely pathogenicity with four predictive tools. This variant was found associated with a second allele. It was absent in healthy control alleles but reported in ESP project (1/4405). Moreover, a known mutation Arg764Cys was reported in the same residue.

A total of 15 novel variants were identified during the current screening of *CRB1*: 4 missense variants, 4 nonsense mutations, 3 frameshift indels and 2 splicing-site mutations (Table 3, Additional file 1: Table S2). Pathogenicity of the novel variants was assessed by co-segregation with a second disease allele on the *CRB1* gene whenever family members were available (Additional file 2: Figures S2, S3 and S4) and by absence in 330 healthy Spanish control chromosomes. None of the novel variants were described as polymorphic changes in the dbSNP database or the literature. All mutations described herein were submitted to the existing Locus Specific Databases (LSDB) on *CRB1*. The novel missense mutations affected conserved residues of the *CRB1* protein (Additional file 2: Figure S5) and were classified as likely damaging by *in silico* prediction tools (Table 3).

## Discussion

Mutations in CRB1 are a common cause of congenital or severe childhood-onset RD accounting for up to 10.1% of LCA/EORD patients and 2.7% in RP cases, as described in a recent meta-analysis of *CRB1* mutations [10]. The above frequencies may be underestimated, as sequencing of entire coding regions had not been systematic performed. In this sense, *CRB1* analysis in large cohorts of patients has been mostly performed by us and other groups using SSCP or chip-based screening for known RD mutations [3-5,8,10,15,16,21]. Only Coppieters et als performed a subsequent an exhaustive screening of *CRB1* and other LCA-related genes by Sanger sequencing in all patients with negative chip results obtaining a higher frequency (16%) of CRB1 mutations [22]. Similarly, we aimed to evaluate herein the real relevance of *CRB1* mutations in the Spanish population using an additional comprehensive mutational screening by HRM and Sanger sequencing, identifying causative variants in 11% of LCA/EORP cases. It is noteworthy that we found a high number of private *CRB1* mutations in our cohort confirming the usefulness of in-depth *CRB1* genetic analysis to identified novel variants undetectable by genotyping microarrays.

Hitherto, few reports have focused on the implication of *CRB1* mutations in RP patients [6][8][10]. *CRB1* defects seemed to explain <3% of EORP cases as described in our previous reports in Spanish patients [15,16] and other population [8,10]. However, it is remarkable that herein up to 9% of our EORP patients carried two *CRB1* mutations. We presumed that this improved detection rate of *CRB1* variants could be due not only to the use of an updated arRP microarray containing some mutations first described in the Spanish population but also for carrying out a comprehensive analysis of the whole gene. Although the most frequent mutation in EORP patients is p.Cys948Tyr occurring worldwide [7], it is noteworthy that 25% of pathogenic *CRB1* alleles correspond to 2 apparently population-specific mutations, the c.498_506del and p.Ile1100Thr variants [10]. In fact, 19 of 25 different EORP-associated *CRB1* mutations, representing 61% of *CRB1* alleles, were first identified in our cohort, that evidences a high frequency of population-specific mutations in the Spanish EORP patients.

Focusing on the LCA patients, *CRB1* defects accounted for 14% of Spanish cases. In view of the high detection rate obtained, APEX genotyping seems to be a quite effective and fast diagnostic approach to detect *CRB1* mutations in our LCA cohort. However, 4 additional patients were identified carrying novel mutations by a further *CRB1* complete screening. The frequency of *CRB1* mutations varies among different studies, ranging from 1% in a cohort of LCA patients hailing from worldwide countries [23] to 16% in Belgium [22]. Although the prevalence of *CRB1* defects is apparently identical between the Belgian and Spanish cohorts, substantial differences are evidenced in the mutational *CRB1* spectrum. The missense variant p.Cys948Tyr is also the most frequent disease allele in Spanish LCA patients, representing 31% of total alleles, but not in Belgium being less prevalent with a frequency of 23% [22]. In contrast, the most recurrent allele in Belgian patients, p.Lys801*, was only detected in one Spanish LCA case. Interestingly, 12 out 17 different LCA-associated *CRB1* variants, representing 44% of total alleles, seem to be specific to our cohort.

Mutations in *CRB1* were previously associated with a wide range of phenotypic manifestations [1-6,23]. Complete loss of function of the CRB1 protein seems to be more related with development of LCA phenotype, while some residual functionality may remain in childhood-onset patients [9]. Nevertheless, a clear genotype–phenotype correlation could not be established since 2 seeming LOF alleles were not only identified in LCA but also EORD in several cohorts [8]. Interestingly, we observed that a combination of 2 null mutations was only found in about 40% of LCA patients but in none of our EORP patients, as showed in Additional file 2: Figure S6. Null alleles were significantly more frequent in LCA that in EORP patients (χ^2^ = 10.2, p<0.001), as previously suggested [8]. However, a similar proportion of LCA and EORP cases carried a combination of null-allele with missense mutation and several LCA patients also presented 2 missense alleles, being p.Cys948Tyr always involved. In consequence, it is not easy to assign a specific allele combination to a particular phenotype, suggesting a strong influence of environmental factors or genetic modifiers on the severity of the disease. In fact, it is likely that the new next-generation sequencing (NGS) technology will help to identify potential genetic variants that modulate LCA and EORP phenotypes associated to *CRB1* defects.

In view of the high frequency of compound heterozygous variants in *CRB1*, we evaluated the HRM technology that is considered a powerful approach to efficiently discriminate heterozygous variants [17], as an alternative to Sanger sequencing. We accurately identified heterozygous variants in all abnormal melting curves (specificity of 100%); however, we also found 4 false negatives thus obtaining a lower sensitivity of 73% for *CRB1* screening by HRM. Several factors such as amplicon size or GC content could influence the sensitivity to detect melting variants and explain the unexpected high number of false negatives in this analysis as opposed to previously reports [24].

This work supports the importance of comprehensive genetic studies in order to ascertain the real prevalence of retinal gene defects in large cohorts of well-clinically phenotyped patients. Main limitations to in-depth genetic analysis of RD are related to the use of expensive and time-consuming techniques. However the recent advances in NGS technologies and their progressive implementation in the clinical diagnosis will help to improve the molecular diagnosis and also to shed light on genetics of retinal dystrophies.

## Conclusions

This study has allowed us to establish gene-specific frequencies in our population. It is worth to note that, to our knowledge, this study represents the most complete mutational screening of *CRB1* in an early-onset RP cohort. Recently, two large studies of the prevalence of *CRB1* mutations have been published; however the whole gene has not been analyzed in most of the cases [6,10]. By contrast, we performed an additional scanning of *CRB1* using an optimized HRM strategy and/or Sanger sequencing. In summary, *CRB1* defects represent a very common cause of LCA and early-onset RP in the Spanish population.

## Competing interests

No conflicting relationship exists for any author.

## Authors’ contributions

MC, SD, AAF and CA drafted the manuscript, MC and CA designed the study, CA and MB coordinated the study, MC and SD carried out the HRM and Sanger sequencing analysis, MC and AAF carried out the homozygosity mapping, AAF, EV and RRA supervised APEX analysis, SB and EV participated in the mutational screening by SSCP and dHPLC, DC carried out the haplotype analysis, IT and BGS carried out ophthalmologic examinations, FBK, MC, AAF and CA contributed to clinical selection of patients. All authors read and approved the final manuscript.

## Supplementary Material

Additional file 1: Table S1 Primers and conditions used for conventional PCR and real-time PCR. * Redesigned primer. Table S2. *In silico* Predictions of Unlikely Pathogenic Non-Synonymous *CRB1* Variants. Novel variants are in bold. Nucleotide numbering is based on RefSeq DNA accession number NM_201253.1. Lam AG: Laminin AG-like domain, EGF: EGF-like domain, Conservation of the amino acid substituted or deleted in 17 species was detailed. HC: Highly conserved, MC: Moderately conserved, WC: Weakly conserved, and NC: non-conserved residue. The amino acid substitution is predicted damaging by if the SIFT score is <= 0.05 and PROVEAN scores is < -2.5. Polyphen predict a non-synonymous variant as benign, possibly damaging, or probably damaging, if score is < 0.2, between 0.2 and 0.85 or > 0.85. GV: Grantham Variation; GD: Grantham distribution. Class C65: most likely pathogenic, Class C0: less likely pathogenic. Table S3. Overview of Likely Pathogenic Mutations Identified in this Study. Click here for file

Additional file 2: Figure S1 Normalized melt curves and difference plots of five *CRB1* variants used for standardization. Blue curves represent wild-type HRM profiles, except for the p.Cys948Tyr variant. (PDF 80 kb) **Figure S2.** Segregation Analysis of *CRB1* Mutations in Characterized Families with Leber Congenital Amaurosis. The *CRB1* genotype of each available family member is represented below the individual symbol being “+” wild type allele, m, m1 and m2 mutated alleles. Haplotype analysis was performed with markers flanking the *CRB1* gene (CEN-D1S408-D1S2757- D1S2816-D1S1660-TEL). Nucleotide numbering reflects cDNA numbering in the reference sequence NM_201253.1, according to journal guidelines (www.hgvs.org/mutnomen). The initiation codon is codon 1 &: Two distinct retinal dystrophies with mutations affecting 2 different genes cosegregated in this family as previously reported by Riveiro-Alvarez, R. 2008. Patient II:2 exhibited Stargardt disease and carried a homozygous mutation (c.5413A>G; p.Asn1805Asp) in *ABCA4*. However, individual IV:4 presented an early-onset RP that is explained by the presence of two *CRB1* mutations. (PDF 67 kb) **Figure S3.** Segregation Analysis of *CRB1* Mutations in Characterized Families with Early-Childhood-Onset RP. The *CRB1* genotype of each available family member is represented below the individual symbol being “+” wild type allele, m, m1 and m2 mutated alleles. Haplotype analysis was performed with markers flanking the *CRB1* gene (CEN-D1S408-D1S2757- D1S2816-D1S1660-TEL). Nucleotide numbering reflects cDNA in the reference sequence NM_201253.1, according to journal guidelines (www.hgvs.org/mutnomen). The initiation codon is +1. (PDF 272 kb) **Figure S4.** Pedigrees and segregation analysis of families segregating both Leber Congenital Amaurosis and Early-Childhood-Onset RP phenotypes. LCA and early-onset RP phenotypes have been found in the same pedigree caused by different combination of distinct *CRB1* alleles. The *CRB1* genotype of each available family member is represented below the individual symbol being “+” wild type allele, m, m1, m2, m3 and m4 mutated alleles. Haplotype analysis was performed with markers flanking the *CRB1* gene (CEN-D1S408-D1S2757- D1S2816-D1S1660-TEL). Nucleotide numbering reflects cDNA numbering in the reference sequence NM_201253.1, according to journal guidelines (www.hgvs.org/mutnomen). The initiation codon is +1. (PDF 39 kb) **Figure S5.** Evolutionary conservation of the novel missense mutation in the *CRB1* protein. We considered the following 18 species: Human, *Chimpanzee* (*Pan troglodytes*), Marmoset (*Callithrix jacchus*), Rhesus Macaque (*Macaca mulatta*), Orangutan (*Pongo abelii*), Gibbon (*Nomascus leucogenys*), Cow (*Bos taurus*), Rat (*Rattus norvegicus*), *Mouse* (*Mus musculus*), Rabbit (*Oryctolagus cuniculus*), Dog (*Canis lupus familiaris*), Horse (*Equus caballus*), Wild boar (*Sus scrofa*), Opossum (*Monodelphis domestica*), Turkey (*Meleagris gallopavo*), Lizard (*Anolis carolinensis*), Frog (*Xenopus tropicalis*), Zebrafish (*Danio rerio*). Residues on a dark blue background are fully conserved between different species. Residues coloured light blue are similar to amino acid residues in other orthologs. (PDF 113 kb) **Figure S6.** Genotype–phenotype correlation of patients with *CRB1* mutations. Frequency of different combination of null alleles, missense variants and in-frame deletions in LCA and EORP patients. Click here for file
