# Supplementary material for: High frequency of CRB1 mutations as cause of Early-Onset Retinal Dystrophies in the Spanish population
Source: Orphanet J Rare Dis. 2013 Feb 5;8:20. doi: 10.1186/1750-1172-8-20 (PMC3637806; doi:10.1186/1750-1172-8-20)
Supplement: Additional file 1: Table S1 — Primers and conditions used for conventional PCR and real-time PCR. * Redesigned primer. Table S2. In silico Predictions of Unlikely Pathogenic Non-Synonymous CRB1 Variants. Novel variants are in bold. Nucleotide numbering is based on RefSeq DNA accession number NM_201253.1. Lam AG: Laminin AG-like domain, EGF: EGF-like domain, Conservation of the amino acid substituted or deleted in 17 species was detailed. HC: Highly conserved, MC: Moderately conserved, WC: Weakly conserved, and NC: non-conserved residue. The amino acid substitution is predicted damaging by if the SIFT score is <= 0.05 and PROVEAN scores is < -2.5. Polyphen predict a non-synonymous variant as benign, possibly damaging, or probably damaging, if score is < 0.2, between 0.2 and 0.85 or > 0.85. GV: Grantham Variation; GD: Grantham distribution. Class C65: most likely pathogenic, Class C0: less likely pathogenic. Table S3. Overview of Likely Pathogenic Mutations Identified in this Study. [file 1750-1172-8-20-S1.docx]

| **Exon** | **Forward Primer (5’-3’)** | **Reverse Primer (5’-3’)** | **PCR Annealing Temperature (ºC)** | **Real Time PCR Annealing Temperature (ºC)** | **Product Size (bp)** |
| --- | --- | --- | --- | --- | --- |
| 1* | CAGCAACACACCAGAGGATG | ATAATAAGCCAGAAATAAACCAG | 55 | 55 | 150 |
| 2a* | GCAGCACAAAGGTCACAAG | TCCTGATGGCAAATACCTCC | 60 | 58 | 400 |
| 2b | GTACAGTGGGACAATCTGTG | TCACCTCTGCTTCTGCCAC | 60 | 60 | 430 |
| 2c | GATGGAATTGATGGTTACTCC | CTTAGAAAGAGCTAACTACACC | 58 | 58 | 211 |
| 3a | GCTCTGGTAAACAAAGCATTG | GAATCCAGGGGCACAGTCG | 58 | 58 | 200 |
| 3b | GACGAATGTTGGTCCCAGC | CAGAGTGGTAAAATAGTTCATG | 60 | 60 | 240 |
| 4 | GAAACAGTATAAAGATATCTGATC | GCTATAAGCGATATGTGTATTC | 55 | 55 | 275 |
| 5* | TAATTCAACACCTTTGACTTAGC | TGCCATAAAATACCAGAAAGTC | 55 | 55 | 280 |
| 6a | ACAAGTAAATTACGTGAAACTTC | AGTGAGGGATGCATGTTCC | 60 | 60 | 281 |
| 6b | ATTCTCCTGGGCTGTACC | GCTATGTTACAAACTGAGCC | 60 | 60 | 212 |
| 6c | GCGATGGCTTCCTGTGGG | TGCCACTCTCCATCGCTGG | 60 | 60 | 235 |
| 6d | CAGGTCAATAATCAGTCAAAGG | CAAACGAAGGTGTGGATGGC | 58 | 58 | 280 |
| 6e | ACCAGTGGGAATGACCAGC | CTGTGGCAGTCACACTGG | 60 | 60 | 225 |
| 6f | CAACCTTGTCAAAGCAGAGG | CTCTGAGGCATGGCACTCC | 60 | 58 | 140 |
| 7a | TTCTCCTCCTCCTCTATTTTG | ACACGGATATATTGATAAGTGC | 58 | 58 | 196 |
| 7b | CTCCATGTTTGTCCGAACGC | TCTTGCTTGTCAGGTAGGC | 60 | 60 | 303 |
| 7c | TCAGTCTTCACAAAACCTAGG | ATAAAGTAAAAGTTTAGCATACAG | 56 | 56 | 308 |
| 8 | CAACATTTTTCTATTTAGTTGCC | CTCAAATGTCGCAACTTAACTG | 56 | 56 | 276 |
| 9a | AATGATCATTACTATTAATAACGG | GTGCCATCATTCACTGACTG | 55 | 55 | 339 |
| 9b | GTGGCAACAGCTTTTATATGC | CATGAACATTTTCAAAGTAAGAG | 56 | 56 | 303 |
| 9c | ATATAAAGGGCCTGCAAGGG | GCTGCAACTCTGTCAGAGC | 56 | 58 | 325 |
| 9d | GAACTCAACATCGATGAATGC | CAGTGATGCAGAGTATAGCTTC | 56 | 56 | 282 |
| 10 | CTTGAATGAGATGAACAAGATG | GAGGAGAAAATGAACTTTGAG | 56 | 58 | 250 |
| 11* | ACTTTTTCTTCCCATTTCAC | ACAACTGGCTCGTCATTCAT | 55 | 55 | 380 |
| 12 | GCCTTTGCTATAGAATTCGC | AGTACAGTCATCACATTCACA | 55 | 55 | 318 |

**Supplemental Table 1.**

| **Exon** | **Nucleotide change** | **Aa change** | **Domain** | **Conservation** | **GD-GV** | **PolyPhen** | | **SIFT** | | **PROVEAN** | | **Remarks** |
| --- | --- | --- | --- | --- | --- | --- | --- | --- | --- | --- | --- | --- |
|  |  |  |  |  | **Prediction** | **Score** | **Prediction** | **Score** | **Prediction** | **Score** | **Prediction** |  |
| 2 | c.484G>A | p.Val162Met | EGF4 | NC | Class CO | 0.006 | Benign | 0.36 | Tolerated | -0.792 | Neutral | Unknown second allele in our report. Methionine in Marmoset, Cow, Mouse, Rabbit and Dog. rs137853138 MAF = 0.002 (dbSNP) |
| 2 | c.614C>T | p.Ile205Thr | EGF5 | MC | Class C0 | 0.813 | Possibly damaging | 0.00 | Affect protein function | -2.727 | Deleterious | Unknown second allele in our report and other studies (Bernal, et al., 2003; Booij, et al., 2005; den Hollander, et al., 2004; Henderson, et al., 2010; Walia, et al., 2010) and no cosegregation was observed in 2 families (Booij, et al., 2005; den Hollander, et al., 2004) |
| 4 | c.866C>T | p.Thr289Met | EGF7 | NC | Class C0 | 0.001 | Benign | 0.10 | Tolerated | -2.160 | Neutral | Unknown second allele in our report and in 1/330 Spanish control chromosomes. |
| 6 | **c.1796T>C** | **p.Leu599Pro** | Lam AG1 | NC | Class C15 | 0.388 | Benign | 0.01 | Affect protein function | -3.034 | Deleterious | Unknown second allele. Proline in Rat |
| 7 | **c.2614G>A** | **p.Ala872Thr** | Lam AG2 | NC | Class C0 | 0.001 | Benign | 0.64 | Tolerated | -0.707 | Neutral | Unknown second allele. Threonine in Frog |
| 8 | c.2681A>G | p.Asn894Ser | EGF13 | MC | Class C0 | 0.003 | Benign | 0.26 | Tolerated | -2.544 | Deleterious | Unknown second allele in our report and also in den Hollander, et al., 2001a, 2004. Seri in Frog and Turkey |
| 8 | c.2714G>A | p.Arg905Gln | EGF13 | NC | Class C0 | 0.166 | Benign | 0.08 | Tolerated | 0.016 | Neutral | Unknown second allele in our report. No cosegregation in den Hollander, et al., 2004 |
| 11 | c.3992 G>A | p.Arg1331His | EGF19 | MC | Class C0 | 0.465 | Possibly damaging | 0.19 | Tolerated | -1.709 | Neutral | Unknown second allele in our report, no cosegregation and allele in controls in den Hollander, et al., 2004 |

**Supplemental Table 2.**

| **No.** | **exon** | **Nucleotide Change** | **AA Change** | **No. Alleles (%)** | **No. Families** | **No. LCA-associated Alleles (%)** | **LCA families** | **No. EORP-associated Alleles (%)** | **EORP Families** |
| --- | --- | --- | --- | --- | --- | --- | --- | --- | --- |
| 1 | 2 | **c.481dupG** | **p.Ala161Glyfs*8** | 3 (3.4) | 2 | 2 (6.2) | 1 | 1 (1.8) | 1 |
| 2 | 2 | **c.498_506del** | **p.Ile167_Gly169del** | 7 (8) | 6 |  |  | 7 (12.5) | 6 |
| 3 | 2 | c.613_619del | p.Ile205Aspfs*13 | 4 (4.5) | 4 | 3 (9.4) | 3 | 1 (1.8) | 1 |
| 4 | 5 | **c.1147_1156del** | **p.C383Sfs*65** | 1 (1.1) | 1 |  |  | 1 (1.8) | 1 |
| 5 | 6 | **c.1604T>C** | **p.Leu535Pro** | 1 (1.1) | 1 | 1 (3.1) | 1 |  | 0 |
| 6 | 6 | **c.1690G>T** | **p.Asp564Tyr** | 3 (3.4) | 3 | 1 (3.1) | 1 | 2 (3.6) | 2 |
| 7 | 6 | **c.1702C>T** |  | 1 (1.1) | 1 |  |  | 1 (1.8) | 1 |
| 8 | 7 | **c.2227delG** | **p.Val743Serfs*11** | 1 (1.1) | 1 | 1 (3.1) | 1 |  | 0 |
| 9 | 7 | c.2234C>T | p.Thr745Met | 6 (6.8) | 5 |  |  | 6 (10.7) | 5 |
| 10 | 7 | **c.2244_2247del** | **p.Ser749del** | 1 (1.1) | 1 | 1 (3.1) | 1 |  | 0 |
| 11 | 7 | c.2290C>T | p.Arg764Cys | 3 (3.4) | 3 |  |  | 3 (5.4) | 3 |
| 12 | 7 | **c.2291G>A** | **p.Arg764His** | 1 (1.1) | 1 |  |  | 1 (1.8) | 1 |
| 13 | 7 | **c.2309G>T** | **p.Gly770Val** | 1 (1.1) | 1 | 1 (3.1) | 1 |  | 0 |
| 14 | 7 | c.2401A>T | p.Lys801* | 2 (2.3) | 1 | 2 (6.2) | 1 |  | 0 |
| 15 | 7 | **c.2416G>T** | **p.Glu806*** | 1 (1.1) | 1 |  |  | 1 (1.8) | 1 |
| 16 | 7 | **c.2465G>A** | **p.Trp822*** | 1 (1.1) | 1 |  |  | 1 (1.8) | 1 |
| 17 | 8 | c.2688T>A | p.Cys896* | 4 (4.5) | 4 | 2 (6.2) | 2 | 2 (3.6) | 2 |
| 18 | 8 | **c.2696 G>C** | **p.Gly899Ala** | 1 (1.1) | 1 |  |  | 1 (1.8) | 1 |
| 19 | 8 | **c.2805dupA** | **p.His935Glnfs*13** | 1 (1.1) | 1 | 1 (3.1) | 1 |  | 0 |
| 20 | 9 | c.2843G>A | p.Cys948Tyr | 19 (21.6) | 16 | 10 (31.2) | 8 | 9 (16.1) | 8 |
| 21 | 9 | **c.3002A>T** | **p.Ile1001Asn** | 3 (3.4) | 3 | 1 (3.1) | 1 | 2 (3.6) | 2 |
| 22 | 9 | **c.3014 A>T** | **p.Asp1005Val** | 1 (1.1) | 1 |  |  | 1 (1.8) | 1 |
| 23 | 9 | **c.3152G>A** | **p.Trp1051*** | 1 (1.1) | 1 | 1 (3.1) | 1 |  | 0 |
| 24 | 9 | **c.3157 A>G** | **p.Met1053Val** | 2 (2.3) | 2 |  |  | 2 (3.6) | 2 |
| 25 | 9 | c.3299T>C | p.Ile1100Thr | 8 (9.1) | 6 | 1 (3.1) | 1 | 7 (12.5) | 5 |
| 26 | 9 | **c.3482A>G** | **p.Tyr1161Cys** | 1 (1.1) | 1 |  |  | 1 (1.8) | 1 |
| 27 | 9 | **c.3607 G>T** | **p.Glu1203*** | 1 (1.1) | 1 |  |  |  | 1 |
| 28 | 9 | **c.3749+1_3749+2delTG** | **Splicing** | 1 (1.1) | 1 |  |  | 1 (1.8) | 1 |
| 29 | 10 | **c.3878+2insT** | **Splicing** | 1 (1.1) | 1 |  |  | 1 (1.8) | 1 |
| 30 | 11 | **c.3988G>T** | **p.Glu1330*** | 3 (3.4) | 3 | 2 (6.2) | 2 | 1 (1.8) | 1 |
| 31 | 11 | **c.4000delG** | **p.Val1334Trpfs*7** | 1 (1.1) | 1 | 1 (3.1) | 1 |  | 0 |
| 32 | 11 | c.4005+1G>A | Splicing | 1 (1.1) | 1 | 1 (3.1) | 1 |  | 0 |
| 33 | 12 | c.4142G>T | p.Pro1381Leu | 1 (1.1) | 1 |  |  | 1 (1.8) | 1 |
| 34 | 12 | **c.4168C>T** | **p.Arg1390*** | 1 (1.1) | 1 |  |  | 1 (1.8) | 1 |
|  |  |  | **TOTAL** | **88 (100)** |  | **32 (100)** |  | **56 (100)** |  |

**Supplemental Table 3.**
