# Supplementary material for: High frequency of CRB1 mutations as cause of Early-Onset Retinal Dystrophies in the Spanish population
Source: Orphanet J Rare Dis. 2013 Feb 5;8:20. doi: 10.1186/1750-1172-8-20 (PMC3637806; doi:10.1186/1750-1172-8-20)
Supplement: Additional file 2: Figure S1 — Normalized melt curves and difference plots of five CRB1 variants used for standardization. Blue curves represent wild-type HRM profiles, except for the p.Cys948Tyr variant. (PDF 80 kb) Figure S2. Segregation Analysis of CRB1 Mutations in Characterized Families with Leber Congenital Amaurosis. The CRB1 genotype of each available family member is represented below the individual symbol being “+” wild type allele, m, m1 and m2 mutated alleles. Haplotype analysis was performed with markers flanking the CRB1 gene (CEN-D1S408-D1S2757- D1S2816-D1S1660-TEL). Nucleotide numbering reflects cDNA numbering in the reference sequence NM_201253.1, according to journal guidelines (www.hgvs.org/mutnomen). The initiation codon is codon 1 &: Two distinct retinal dystrophies with mutations affecting 2 different genes cosegregated in this family as previously reported by Riveiro-Alvarez, R. 2008. Patient II:2 exhibited Stargardt disease and carried a homozygous mutation (c.5413A>G; p.Asn1805Asp) in ABCA4. However, individual IV:4 presented an early-onset RP that is explained by the presence of two CRB1 mutations. (PDF 67 kb) Figure S3. Segregation Analysis of CRB1 Mutations in Characterized Families with Early-Childhood-Onset RP. The CRB1 genotype of each available family member is represented below the individual symbol being “+” wild type allele, m, m1 and m2 mutated alleles. Haplotype analysis was performed with markers flanking the CRB1 gene (CEN-D1S408-D1S2757- D1S2816-D1S1660-TEL). Nucleotide numbering reflects cDNA in the reference sequence NM_201253.1, according to journal guidelines (www.hgvs.org/mutnomen). The initiation codon is +1. (PDF 272 kb) Figure S4. Pedigrees and segregation analysis of families segregating both Leber Congenital Amaurosis and Early-Childhood-Onset RP phenotypes. LCA and early-onset RP phenotypes have been found in the same pedigree caused by different combination of distinct CRB1 alleles. The CRB1 genotype of each available family member is represente [file 1750-1172-8-20-S2.pdf]

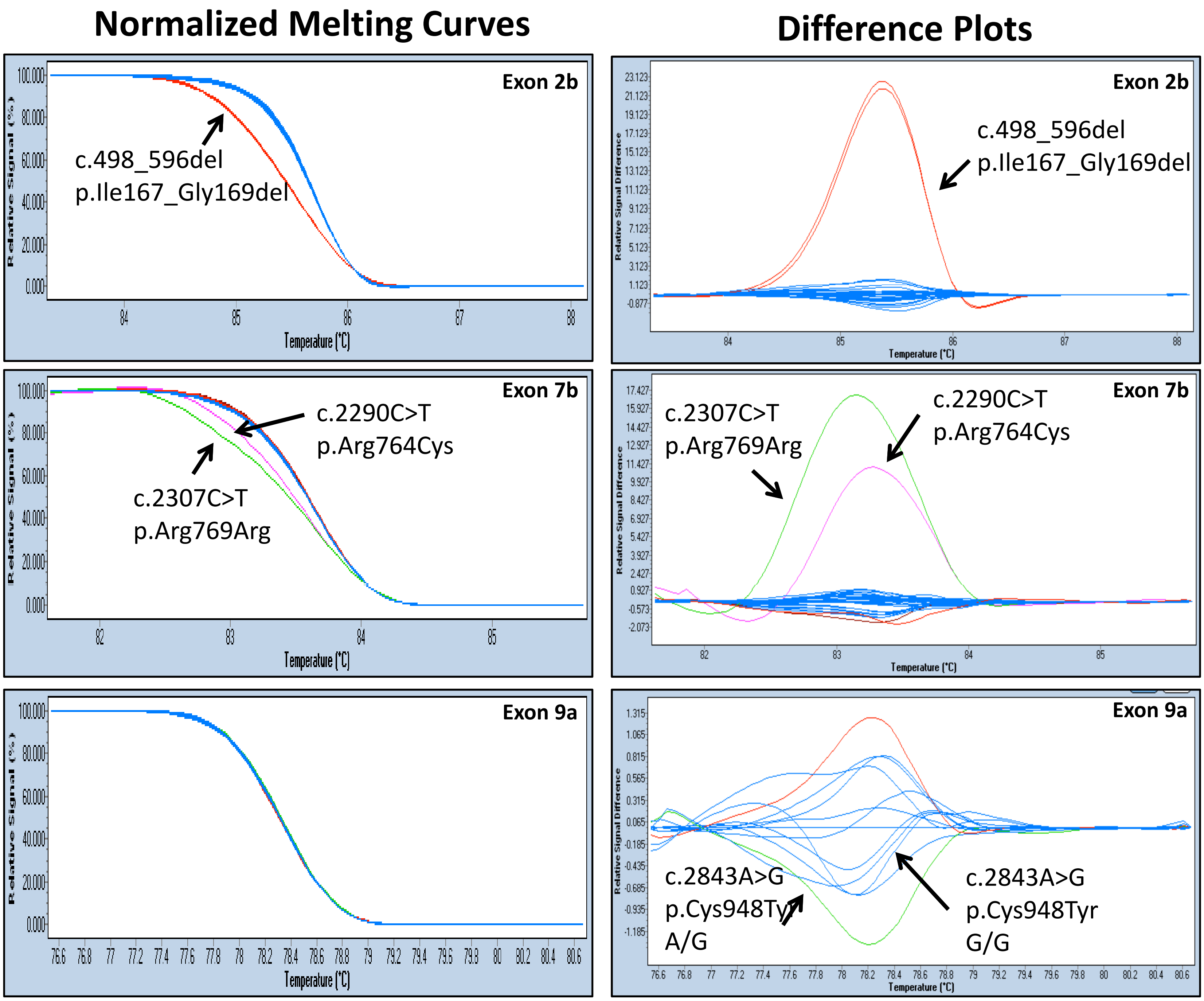
Normalized Melting Curves
Difference Plots
Exon 2b
c.498_596del
p.Ile167_Gly169del
Exon 7b
c.2290C>T
p.Arg764Cys
c.2307C>T
p.Arg769Arg
Exon 9a
c.2843A>G
p.Cys948Tyr
A/G
c.2843A>G
p.Cys948Tyr
G/G
Relative Signal (%)
Relative Signal Difference
Temperature (°C)

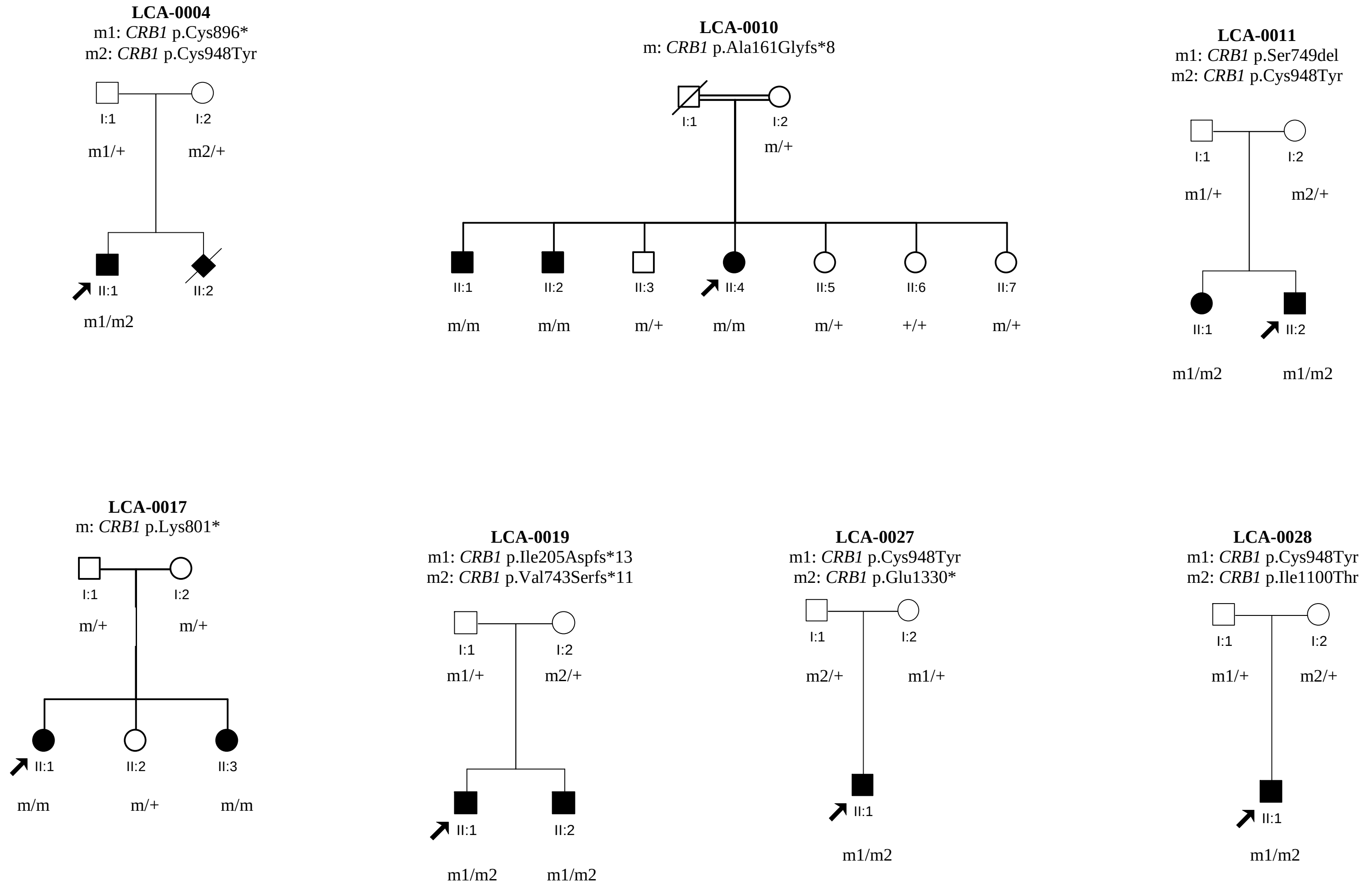

Supplemental Figure S2.

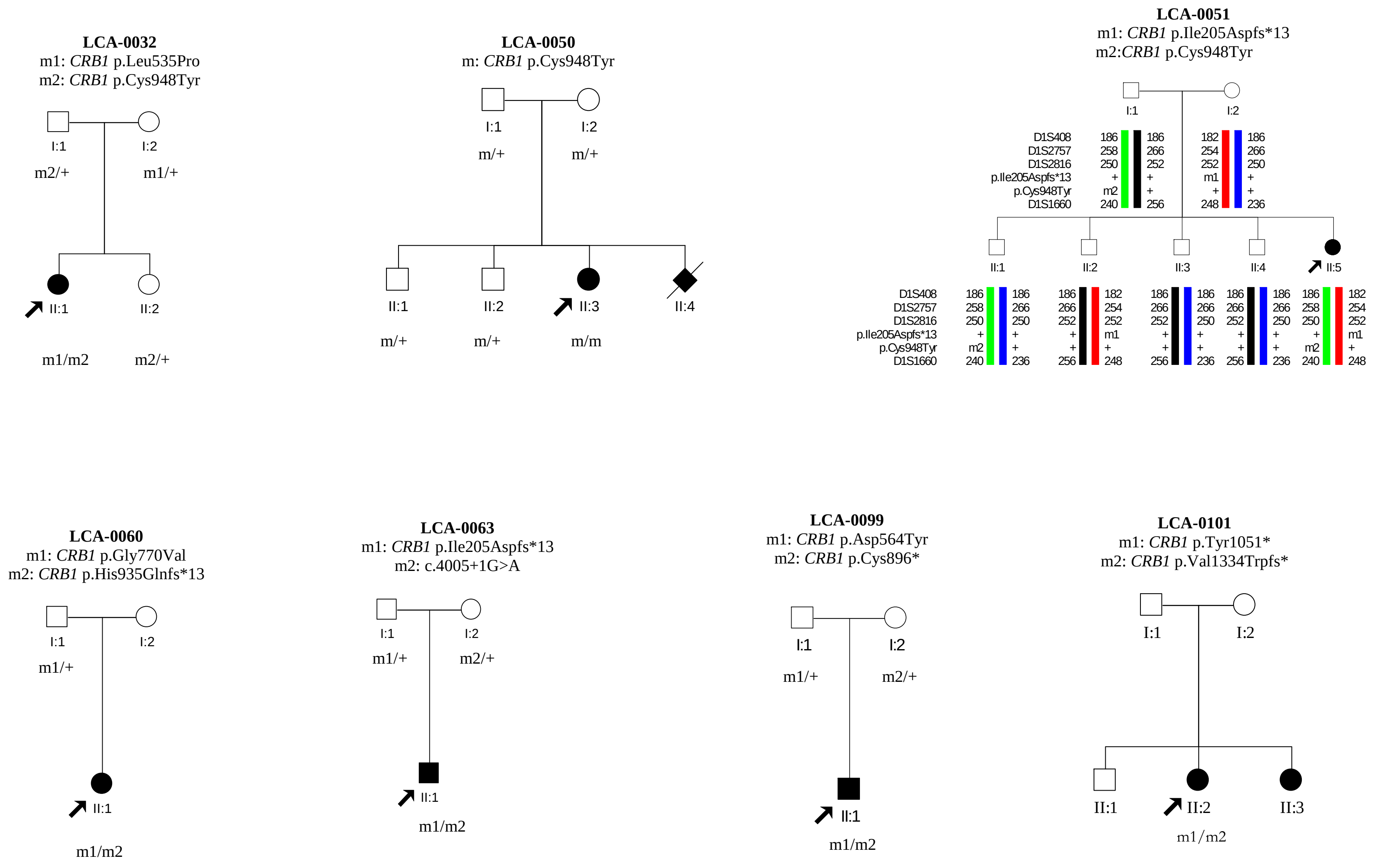

Supplemental Figure S2 (Continued).

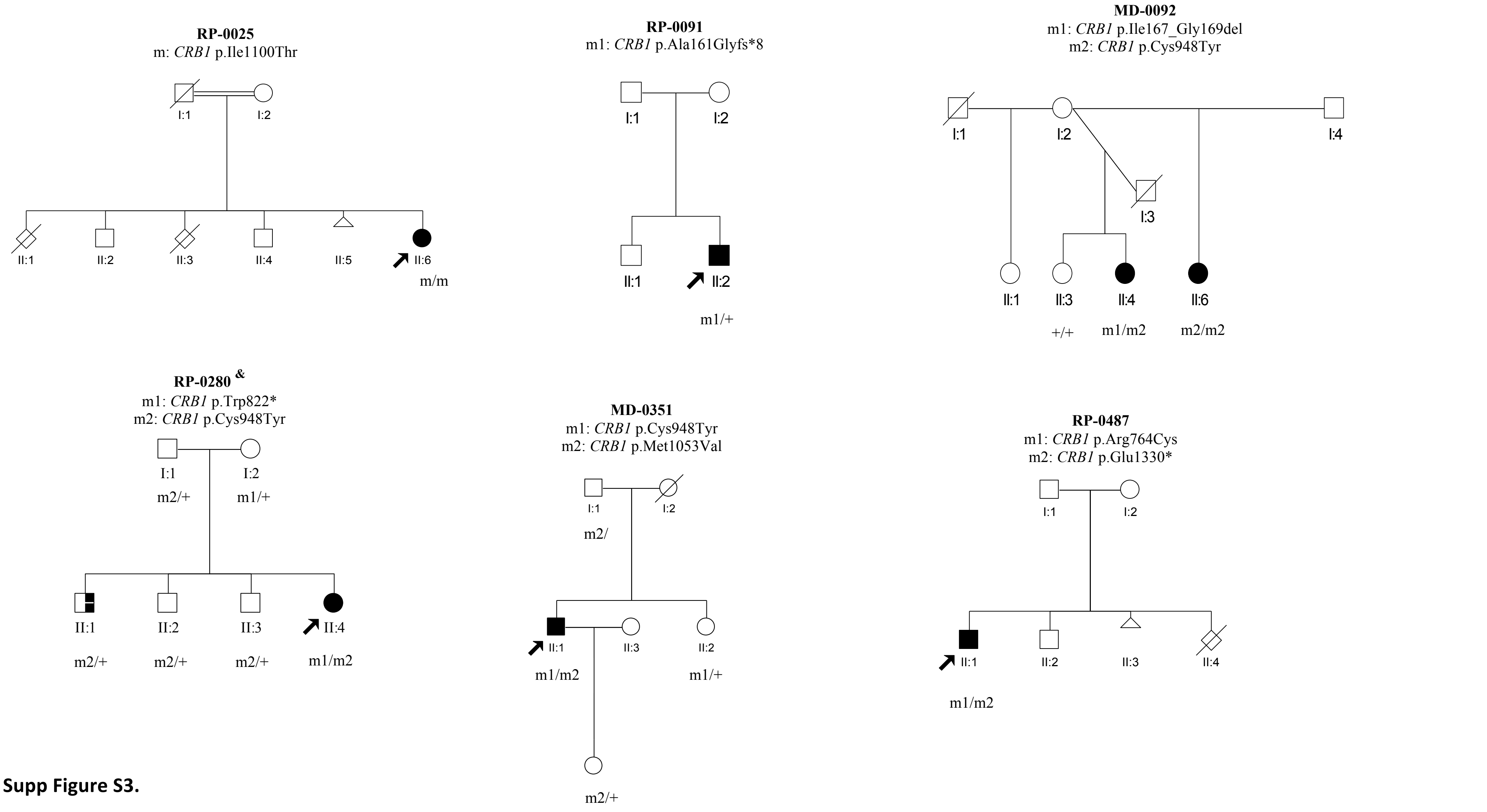

Supp Figure S3.

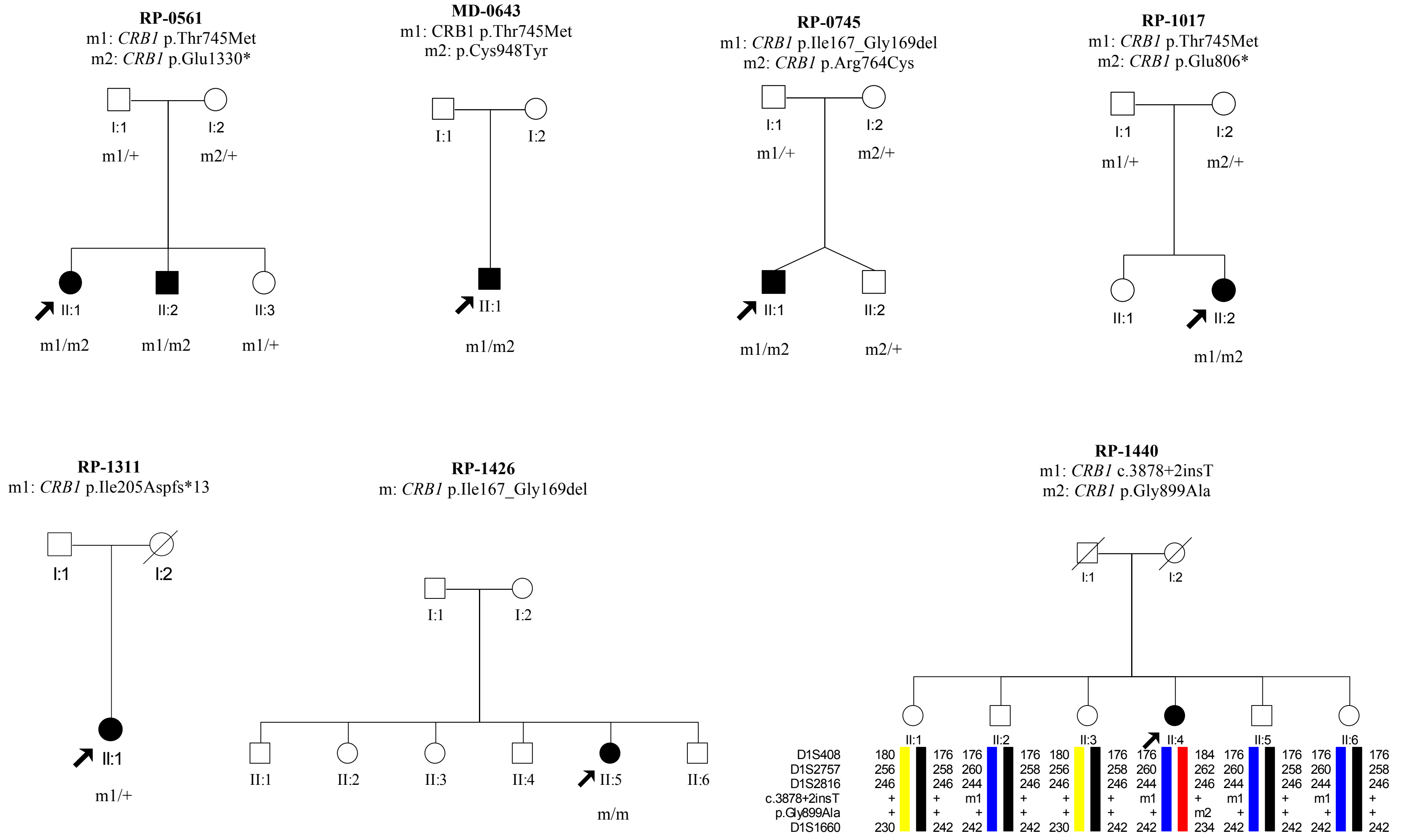

Supp Figure S3. (Continued).

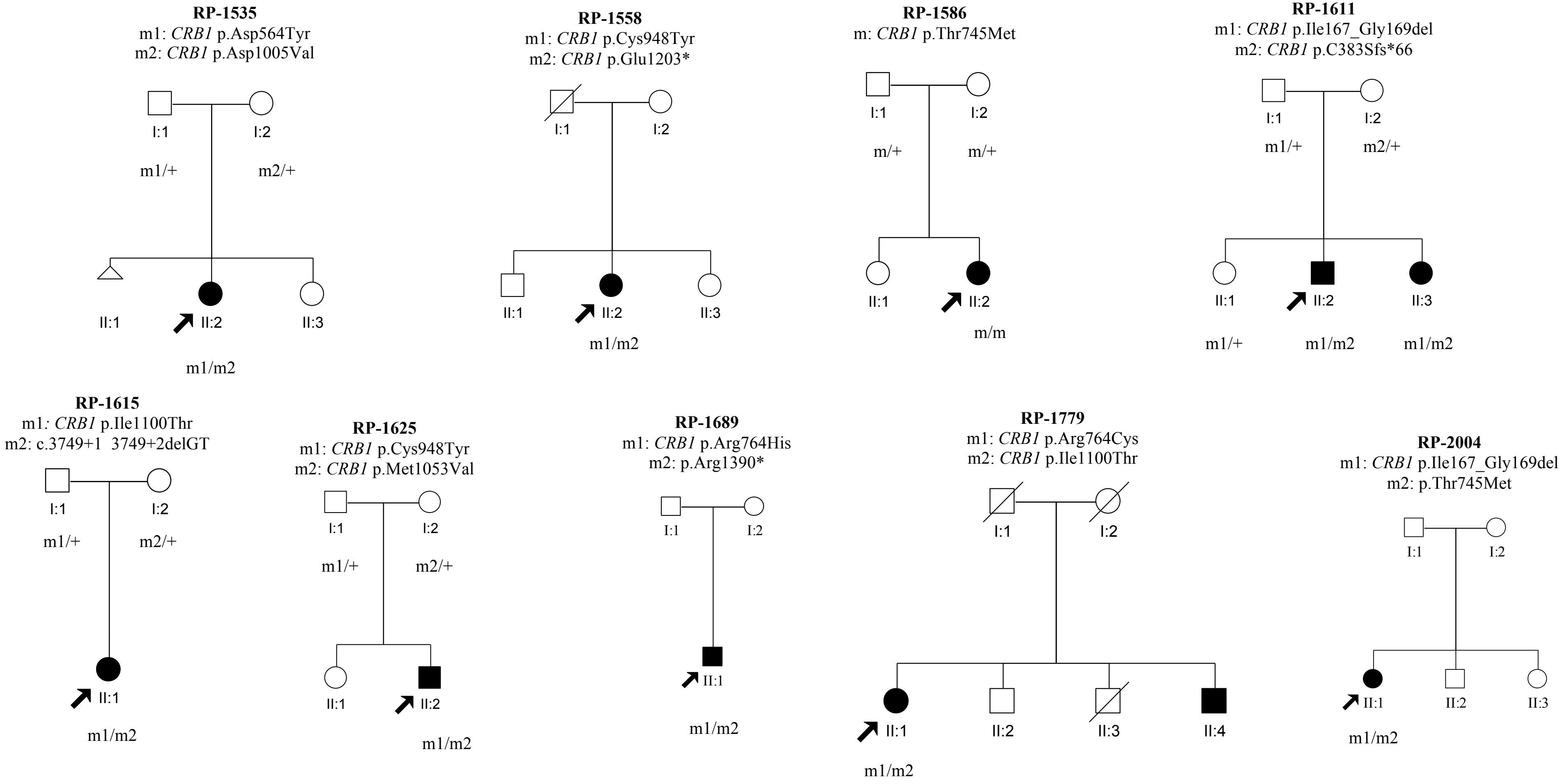

Supp Figure S3. (Continued).

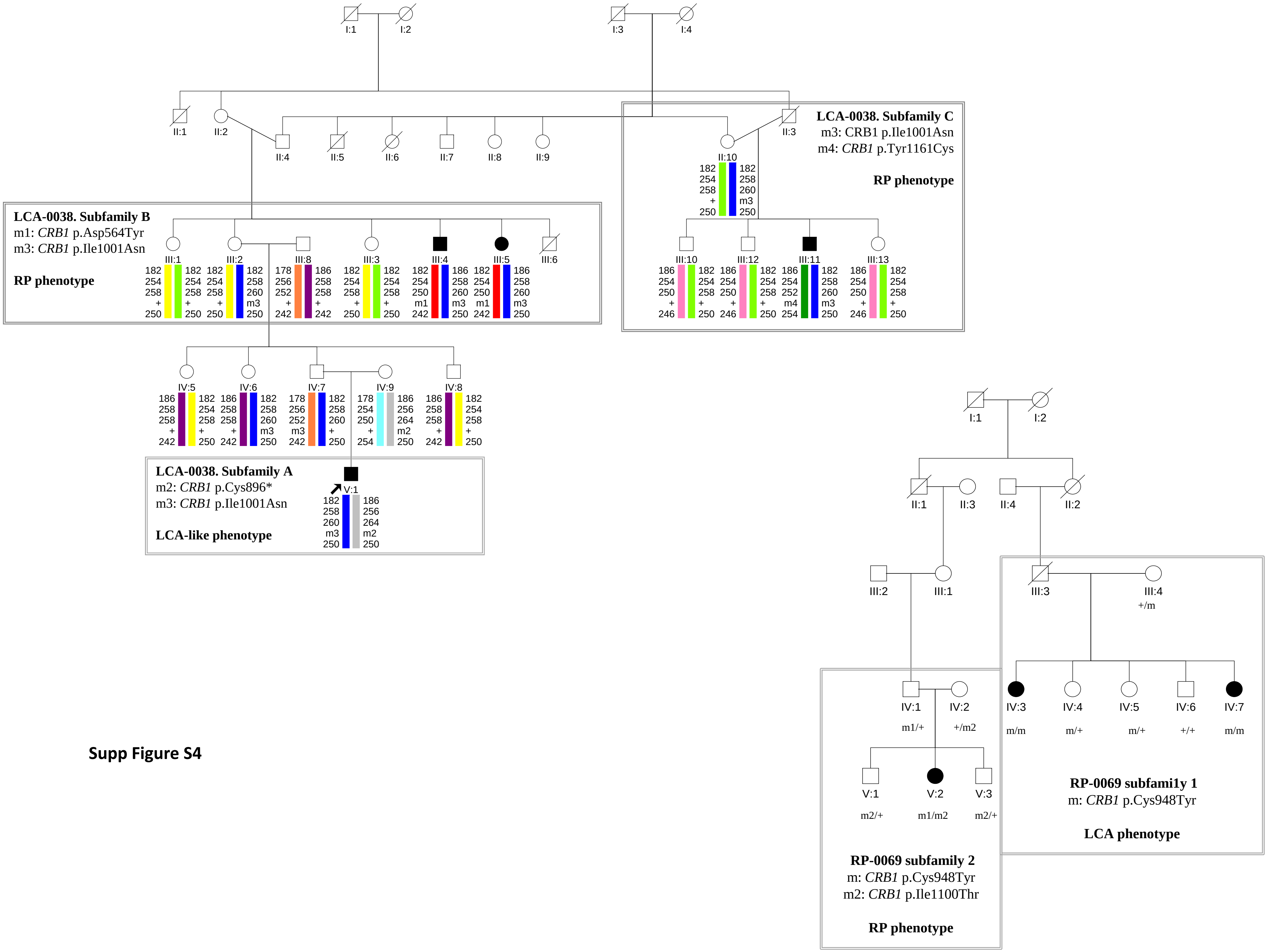

Supp Figure S4

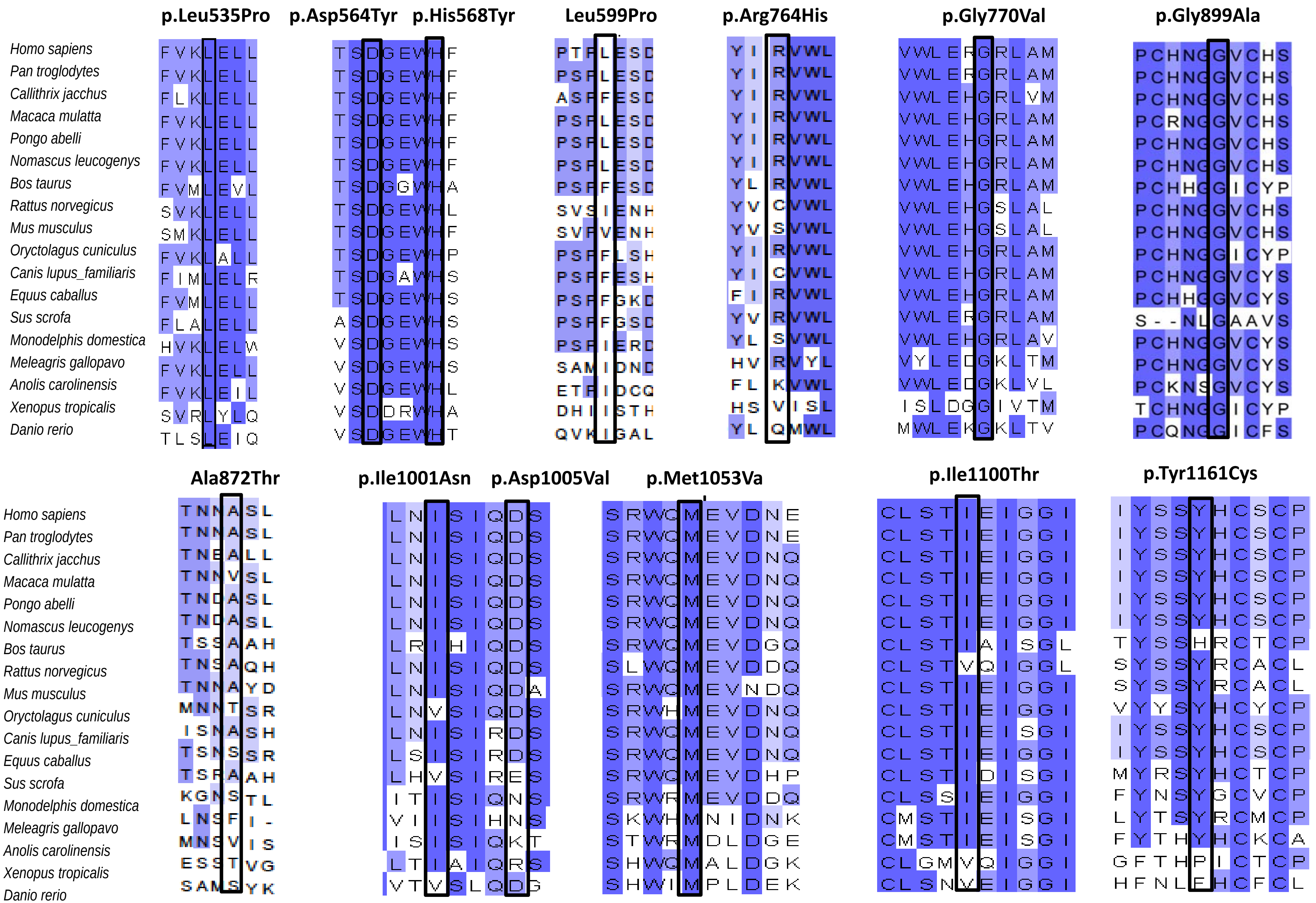

Supp Figure S5

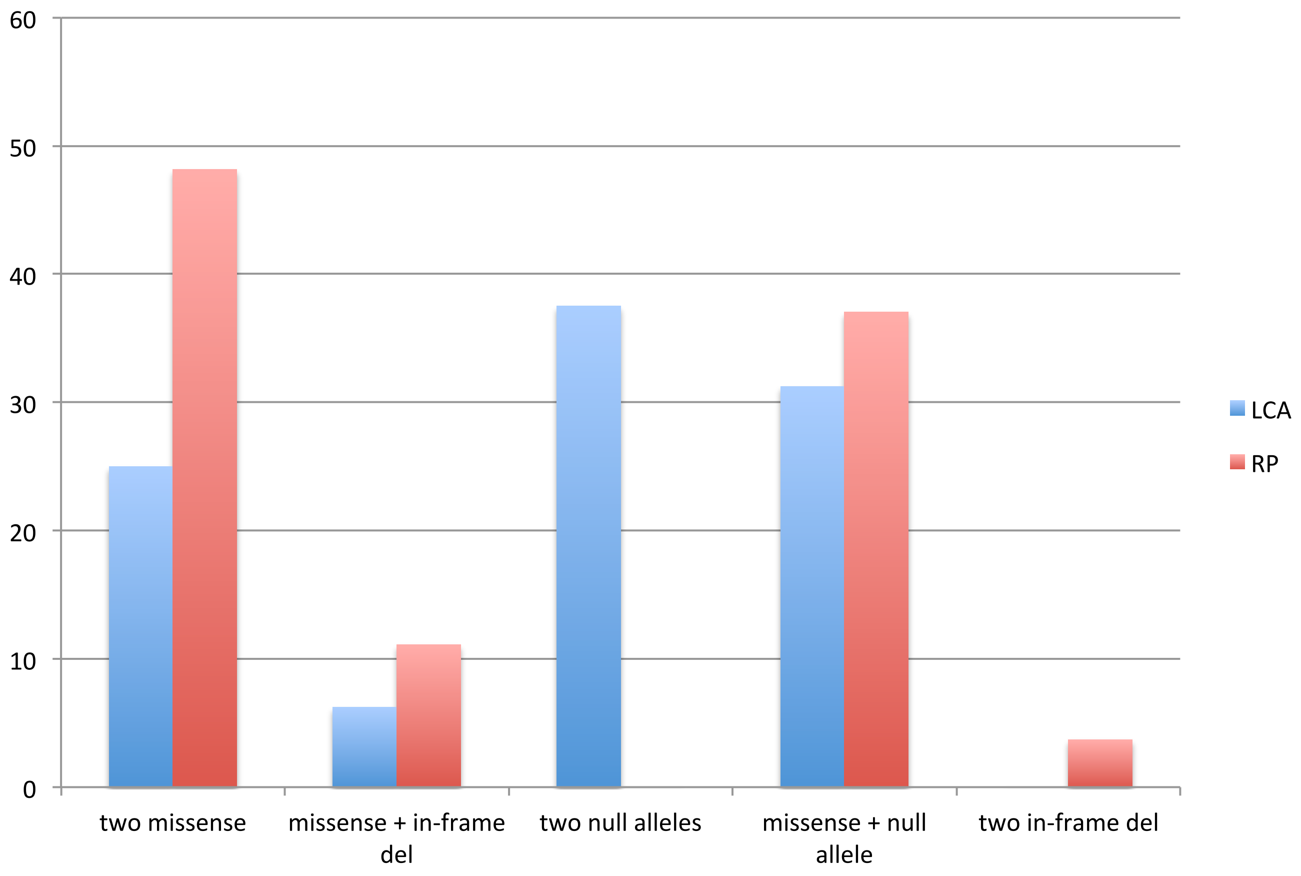

Suppl Figure S6
